# Notice of duplicate publication: Qualitative research and pain: Current controversies and future directions

**DOI:** 10.1080/24740527.2020.1826282

**Published:** 2020-09-30

Tutelman PR, Webster F. Qualitative research and pain: Current controversies and future directions. Can J Pain. 2020. doi:10.1080/24740527.2020.1814131.

Please note that this article has been removed from *Canadian Journal of Pain* as it is a duplicate publication of:

Tutelman PR, Webster F. Qualitative research and pain: Current controversies and future directions. Can J Pain. 2020;4(3): 1–5. doi:10.1080/24740527.2020.1809201.

